# β-Hemolysis May Not Be a Reliable Indicator of Leukotoxicity of *Mannheimia haemolytica* Isolates

**DOI:** 10.3390/toxins10050173

**Published:** 2018-04-25

**Authors:** Jegarubee Bavananthasivam, Sudarvili Shanthalingam, Abirami Kugadas, Bindu Raghavan, Sai Batra, Subramaniam Srikumaran

**Affiliations:** Department of Veterinary Microbiology and Pathology, College of Veterinary Medicine, Washington State University, Pullman, WA 99164-7040, USA; jbavanan@uoguelph.ca (J.B.); sudar@vetmed.wsu.edu (S.S.); abirami@vetmed.wsu.edu (A.K.); bindu@vetmed.wsu.edu (B.R.); sai@vetmed.wsu.edu (S.B.)

**Keywords:** *Mannheimia haemolytica*, leukotoxin, leukotoxicity, hemolysis, indicator

## Abstract

*Mannheimia* (*Pasteurella*) *haemolytica* causes bronchopneumonia in domestic and wild ruminants. Leukotoxin is the critical virulence factor of *M. haemolytica*. Since β-hemolysis is caused by a large number of leukotoxin-positive *M. haemolytica* isolates, all β-hemolytic *M. haemolytica* isolates are considered to be leukotoxic as well. However, conflicting reports exist in literature as to the leukotoxic and hemolytic properties of *M. haemolytica*. One group of researchers reported their leukotoxin-deletion mutants to be hemolytic while another reported their mutants to be non-hemolytic. The objective of this study was to determine whether β-hemolysis is a reliable indicator of leukotoxicity of *M. haemolytica* isolates. Ninety-five isolates of *M. haemolytica* were first confirmed for presence of leukotoxin gene (*lkt*A) by a leukotoxin-specific PCR assay. Culture supernatant fluids from these isolates were then tested for presence of leukotoxin protein by an ELISA, and for leukotoxic activity by a cytotoxicity assay. All isolates were tested for β-hemolysis by culture on blood agar plates. Sixty-two isolates (65%) produced leukotoxin protein while 33 isolates (35%) did not. Surprisingly, 18 of the 33 isolates (55%), that did not produce leukotoxin protein, were hemolytic. Of the 62 isolates that produced leukotoxin, 55 (89%) were leukotoxic while 7 (11%) were not. All except one of the 55 leukotoxic isolates (98%) were also hemolytic. All seven isolates that were not leukotoxic were hemolytic. Taken together, these results suggest that β-hemolysis may not be a reliable indicator of leukotoxicity of *M. haemolytica* isolates. Furthermore, all *M. haemolytica* isolates that possess *lkt*A gene may not secrete active leukotoxin.

## 1. Introduction

*Mannheimia* (*Pasteurella*) *haemolytica* is a Gram-negative coccobacillus that causes bronchopneumonia in domestic and wild ruminants [1,2,3]. The virulence factors of *M. haemolytica* include the capsule, outer membrane proteins, lipopolysaccharide and leukotoxin (Lkt) [4]. *M. haemolytica* Lkt belongs to the family of Gram-negative bacterial exotoxins, referred to as the RTX (repeats in toxin) toxins. It shares extensive homology with the exotoxins produced by *Escherichia coli* [5], *Actinobacillus pleuropneumoniae* [6], and *Actinobacillus actinomycetemcomitans* [7]. However, leukotoxic activity of *M. haemolytica* Lkt is specific for ruminant leukocytes [8,9]. Although all subsets of ruminant leukocytes are susceptible to Lkt-induced cytolysis, polymorphonuclear cells (PMNs) are the most susceptible subset [10]. Based on the finding that Lkt-deletion mutants cause no mortality [11] or reduced mortality and much milder lung lesions [12,13], Lkt has been widely accepted as the critical virulence factor of *M. haemolytica*. Lkt-induced lysis and degranulation of PMNs is primarily responsible for the acute inflammation and lung injury characteristic of this disease [14,15,16]. Since a large number of leukotoxic *M. haemolytica* isolates are β-hemolytic, β-hemolytic *M. haemolytica* isolates have been broadly assumed to be leukotoxic as well. However, there are conflicting reports in the literature as to the leukotoxic and hemolytic properties of *M. haemolytica*. One group of researchers reported their leukotoxin-deletion mutants to be hemolytic [17] while others reported their mutants to be non-hemolytic [18,19]. The objective of this study, therefore, was to determine whether β-hemolysis is a reliable indicator of leukotoxicity of *M. haemolytica* isolates.

## 2. Results and Discussion

### 2.1. All M. haemolytica Isolates that Possess the lktA Gene May Not Produce Lkt Protein

**Figure 1:**
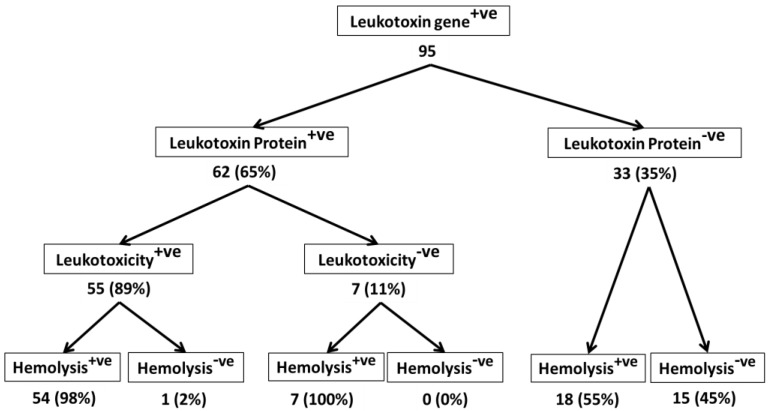
Leukotoxic and hemolytic activity of *M. haemolytica* isolates from bighorn sheep, domestic sheep and cattle. A total of 95 *M. haemolytica* isolates (65 from bighorn sheep and 15 each from domestic sheep and cattle) were analyzed. Presence of leukotoxin gene in *M. haemolytica* isolates was determined by an *lkt*A-specific PCR assay [23]. Presence of leukotoxin protein in the culture supernatant fluids was determined by an indirect ELISA [24]. Leukotoxicity of the isolates was determined by the MTT dye-reduction cytotoxicity assay [25] of culture supernatant fluids on PMNs from the respective species. β-hemolytic activity of the isolates was determined by the standard hemolysis assay [26]. Each isolate was tested three times by each of the assays.

The ELISA used to detect Lkt protein revealed that Lkt protein was not present in the culture supernatant fluids from 33 (35%) of the 95 isolates (Figure 1). Lkt is encoded by the *lktCABD* operon where *lktA* encodes the inactive protoxin, *lktC* encodes a trans-acylase that adds fatty acid chains to internal lysine residues in the protoxin, and *lktB* and *lktD* encoded components of a type 1 secretion system apparatus, along with the outer membrane protein TolC, secrete the toxin from the cell [5,20,21]. LktC-mediated acylation of LktA protoxin is not required for its expression or secretion [22]. It is plausible that in the isolates that were negative for Lkt protein, the secretion system apparatus was defective or totally absent. It is also possible that although the *lktA* gene was present, it was not functional. Further experiments are necessary to determine whether the Lkt protein was not synthesized, or the synthesized protein was not secreted, in these isolates whose culture supernatant fluids were negative for the Lkt protein by ELISA. These experiments are beyond the scope of this study.

### 2.2. Lkt-Negative M. haemolytica Isolates Can Induce Hemolysis

More importantly, 18 (55%) of the 33 isolates that did not have Lkt protein in the culture supernatant fluid were hemolytic (Figure 1), suggesting that not all hemolytic isolates cause leukotoxicity to target cells. Thus, hemolysis may not be a reliable indicator of leukotoxicity of *M. haemolytica* isolates. In this context, it is interesting to note that Miller et al. [27] showed that 16 isolates of the closely related bacterium *Bibersteinia trehalosi* that were reported to be hemolytic, were *lktA* gene negative.

### 2.3. Not All Lkt-Positive M. haemolytica Isolates May Cause Leukotoxicity

Of the 62 isolates that were positive for the presence of Lkt protein in the culture supernatant fluid, only 55 (89%) induced leukotoxicity to target cells, while 7 (11%) did not (Figure 1). As mentioned earlier, Lkt structural protein is encoded by *lkt*A gene as an inactive protoxin, which is converted to biologically active Lkt by the *lktC*-encoded trans-acylase that adds fatty acid chains to internal lysine residues in the protoxin. It is likely that in some isolates, *lktC gene* is defective or totally absent. Reports of development of mutants that produce inactive Lkt by inactivation of *lktC gene* [28] supports this notion. However, further experiments are necessary to confirm this notion.

### 2.4. Majority of Leukotoxic M. haemolytica Isolates Are Hemolytic

Of the 55 isolates that were leukotoxic, 54 (98%) were hemolytic while one (2%) was not. These results suggest that leukotoxic isolates are for the most part hemolytic, but hemolytic isolates may not necessarily be leukotoxic as well. This notion is also supported by the fact that all 7 non-leukotoxic isolates were hemolytic (Figure 1).

**Figure 2:**
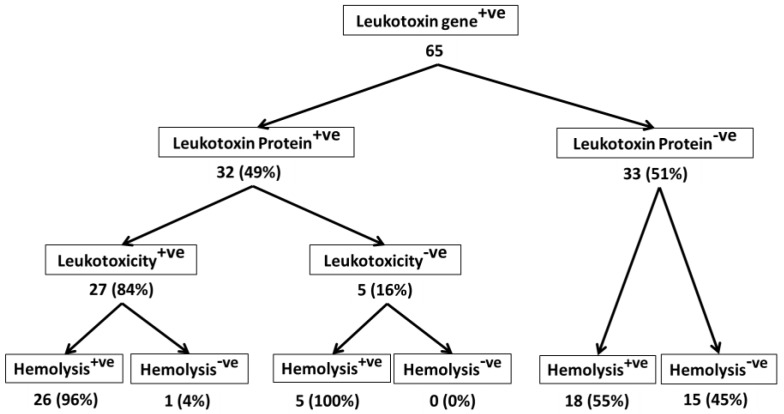
Leukotoxic and hemolytic activity of bighorn sheep isolates of *M. haemolytica*. This figure shows the leukotoxic and hemolytic activities of 65 bighorn sheep isolates of *M. haemolytica* that were part of the 95 isolates shown in Figure 1.

**Figure 3:**
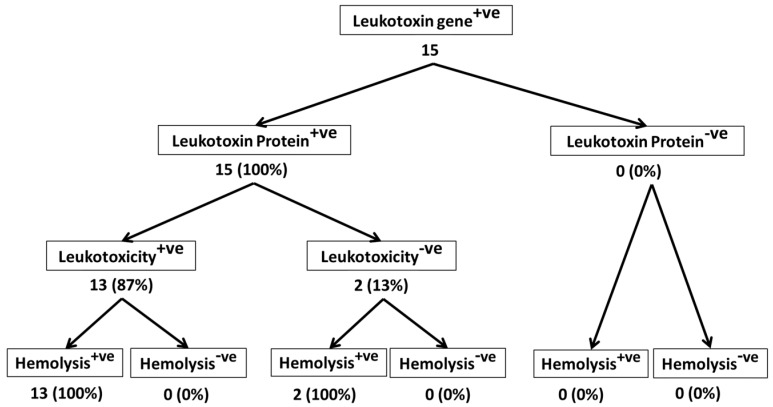
Leukotoxic and hemolytic activity of domestic sheep isolates of *M. haemolytica.* This figure shows the leukotoxic and hemolytic activities of 15 domestic sheep isolates of *M. haemolytica* that were part of the 95 isolates shown in Figure 1.

**Figure 4:**
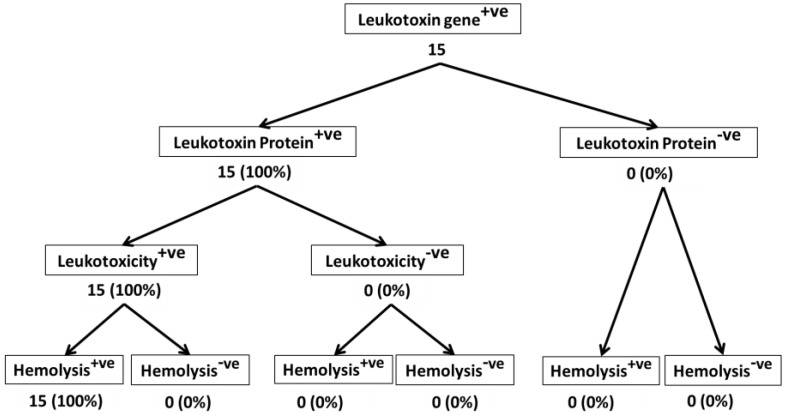
Leukotoxic and hemolytic activity of cattle isolates of *M. haemolytica*. This figure shows the leukotoxic and hemolytic activities of 15 cattle isolates of *M. haemolytica* that were part of the 95 isolates shown in Figure 1.

Of the 95 isolates that were analyzed in this study, 65 were from bighorn sheep, and 15 each were from domestic sheep and cattle. Differences among the isolates from bighorn sheep, domestic sheep, and cattle are apparent when we consider the % of *lktA* gene-positive isolates that secreted Lkt protein, the % of Lkt protein-positive isolates that induced leukotoxicity, and the % of leukotoxic isolates that are hemolytic (Figure 2, Figure 3 and Figure 4). While only 49% of *lktA* gene-positive isolates from bighorn sheep secreted Lkt protein, 100% of the isolates from domestic sheep and cattle secreted Lkt protein. The difference among the three species in the % of isolates that were leukotoxic is small (84%, 87%, and 100% for bighorn sheep, domestic sheep and cattle, respectively). The % of leukotoxic isolates that were hemolytic was very similar in the three species (96%, 100%, and 100% for bighorn sheep, domestic sheep and cattle, respectively). Nevertheless, it should be noted that the number of isolates from domestic sheep and cattle (15 each) analyzed in this study is much smaller than the number of isolates from bighorn sheep (65). When larger number of isolates from domestic sheep and cattle are analyzed, it is very likely that similar results will be observed for the isolates from the different species. Nevertheless, the findings of this study suggest that there may be no correlation between hemolysis and leukotoxicity of *M. haemolytica* isolates.

Leukotoxin cytotoxicity assay is relatively more laborious than hemolysis assay. Probably because of this reason, most laboratories routinely perform only hemolysis assay on *M. haemolytica* isolates. Isolates that are β-hemolytic are considered to be leukotoxic as well. More recently, *lkt*A PCR assay has been included as a means of identifying leukotoxic isolates of *M. haemolytica*. *lkt*A gene-positive isolates are considered to be leukotoxic. The findings of this study suggest that hemolysis may not be a reliable indicator of leukotoxicity of *M. haemolytica* isolates. This study also suggests that *lkt*A PCR assay may not be substituted for cytotoxicity assays for determination of leukotoxicity of *M. haemolytica* isolates.

The findings of this study raise the question as to what component of *M. haemolytica* causes hemolysis. Unfortunately, no information is available on *M. haemolytica* hemolysin protein or the gene that encodes it. We are hopeful that our findings will prompt studies aimed at the elucidation of the hemolytic activity of *M. haemolytica*.

## 3. Conclusions

1. Not all isolates of *M. haemolytica* that possess the *lkt*A gene express the Lkt protein; 2. Not all secreted Lkt exhibit leukotoxic activity; 3. Majority of leukotoxic isolates of *M. haemolytica* are hemolytic, but not all hemolytic isolates of *M. haemolytica* are leukotoxic; 4. β-hemolysis may not be a reliable indicator of leukotoxicity of *M. haemolytica* isolates; 5. It is prudent to determine the leukotoxicity of the culture supernatant fluid by cytotoxicity assay with target cells before concluding an isolate of *M. haemolytica* to be leukotoxic.

## 4. Materials and Methods

### 4.1. Experimental Design

Ninety-five field isolates of *M. haemolytica* [65 from bighorn sheep (*Ovis canadensis*) and 15 each from domestic sheep (*Ovis aries*) and cattle (*Bos taurus*)] were used in this study. All were revived from freezer stock on brain heart infusion (BHI) agar plates. Single colonies were sub-cultured. All isolates were first confirmed to be *bona fide M. haemolytica* isolates by a species-specific PCR assay [23]. Subsequently, they were tested for the presence of Lkt gene (*lkt*A) by an Lkt-specific PCR assay. Culture supernatant fluids from these isolates were then tested for the presence of Lkt protein by an ELISA, and for leukotoxic activity by a cytotoxicity assay. All isolates were tested for β-hemolysis by culture on blood agar plates.

### 4.2. M. haemolytica and Lkt-Specific PCR Assays

The *M. haemoltyica*-specific PCR assay, and the Lkt-specific PCR assay were performed as previously described [23]. Briefly, the forward and reverse primers for the *M. haemoltyica*-specific PCR assay were TGG GCA ATA CGA ACT ACT CGG G, and CTT TAA TCG TAT TCG CAG, respectively. For the Lkt-specific PCR assay, the forward and reverse primers were CTT ACA TTT TAG CCC AAC GTG and TAA ATT CGC AAG ATA ACG GG, respectively. PCR assay consisted of an initial denaturation step at 95 °C for 5 min and a final extension at 72 °C for 10 min for both PCR assays. Cycling conditions used were 40 cycles of 95 °C for 30 s, 54 °C for 30 s, and 72 °C for 30 s for *M. haemolytica* PCR, and 95 °C for 30 s, 52 °C for 30 s, and 72 °C for 40 s for Lkt PCR.

### 4.3. Leukotoxin Production

Leukotoxin from *M. haemolytica* was produced as described previously [25]. Briefly, bacteria were grown to logarithmic phase in BHI broth (Remel, Lenexa, KS, USA) at 37 °C, harvested by centrifugation (13,500× *g* for 20 min at 4 °C), and resuspended in twice the original culture volume of colorless RPMI 1640 medium (Invitrogen, Carlsbad, CA, USA). After an additional 1–1.5 h of growth at 37 °C, the bacteria were pelleted by centrifugation, and the supernatant fluid was filter-sterilized using a 0.22 μm filter. This supernatant preparation containing Lkt was stored at −20 °C until needed.

### 4.4. ELISA for Detection of Lkt Protein

Presence of Lkt protein in bacterial culture supernatant fluids was determined by an indirect ELISA described previously [24], with minor modifications. Lkt in supernatant fluid immobilized on the ELISA plate wells was detected by the addition of a Lkt-specific mouse monoclonal antibody, MM601 [25]. Binding of the mouse monoclonal antibody was detected by the addition of HRP-conjugated sheep anti-mouse Ig antibodies followed by the substrate ABTS. The supernatant fluids that were negative by this ELISA with MM601 were re-confirmed to be negative by an ELISA using a bighorn sheep polyclonal serum known to detect Lkt.

### 4.5. Assay for Detection of Leukotoxic Activity

Leukotoxic activity in culture supernatant fluids was determined by the MTT [3-(4,5-dimethylthiazoyl-2-yl)-2,5-diphenyl tetrazolium bromide] dye-reduction cytotoxicity assay described previously [25]. Briefly, the supernatant fluid was incubated with PMNs from bighorn sheep, domestic sheep, or cattle, and subsequently the viability of the PMNs was determined by the addition of MTT dye which gets converted to a purple formazan precipitate by ER-resident enzymes in viable cells. The O.D of the acid isopropanol-dissolved formazan precipitate is directly proportional to the viability of the cells. The % cytotoxicity was calculated by the following formula: % cytotoxicity = [1 − (OD of toxin-treated cells/OD of toxin-untreated cells)] × 100.

### 4.6. Assay for Detection of β-Hemolysis

The hemolytic ability of *M. haemolytica* isolates was detected by the standard hemolysis assay [26]. *M. haemolytica* isolates were plated on Columbia blood agar plates containing 5% sheep blood (Carolina Biological Supply Company, Burlington, NC, USA) and incubated overnight. The following day, the plates were observed for β-hemolysis with transmitted light. Non-hemolyic isolates were incubated further for another 24 h and observed again for the hemolytic zone. Hemolysis of *M. haemolytica* isolates was determined by comparison with the hemolysis of *Staphylococcus aureus* and *Streptococcus pneumoniae*.
